# A precision agriculture solution for water stress estimation in Hass avocado farms in Colombia

**DOI:** 10.1038/s41598-024-82344-4

**Published:** 2024-12-28

**Authors:** Daniel Enrique Medina, Juan David Medina, Julio Alexis Zorro, Daniel Medina Tobon, Juan Jose Gomez, Luis Felipe Giraldo

**Affiliations:** 1 Research and Development Ozmidia SAS, Bogotá, Colombia; 2 Research and Development Xemog, LLC, Apopka, USA; 3 Department of Biomedical Engineering, University of Los Andes, Bogotá, Colombia

**Keywords:** Precision agriculture, Hass avocado, Space-based internet of things, Evapotranspiration, Water stress, Irrigation scheduling, Small and medium farmers, In-ground sensors, Industry 4.0, Climate-change mitigation, Electrical and electronic engineering

## Abstract

Agriculture 4.0 technologies continue to see low adoption among small and medium-sized farmers, primarily because these solutions often fail to account for the specific challenges of rural areas. In this work, we propose and implement a design methodology to develop a Precision Agriculture solution aimed at assisting farmers in managing water stress in Hass avocado crops. This methodology provides a structured approach for development, enabling the identification of key issues and appropriate solutions. The resulting device measures essential weather variables for calculating crop evapotranspiration and effective precipitation, operates without requiring internet or electricity connections, and transmits data globally via satellite connectivity, overcoming the limitations of existing solutions for this crop. As a result, it can detect water stress and provide crucial information for irrigation scheduling. The proposed solution was tested at a working Hass avocado farm for over a year, collecting weather data and undergoing both major and minor revisions during the iterative testing process. The collected data—covering air temperature, relative humidity, sunshine duration, and rainfall—has been made freely available to support further research and development.

**Supplementary Information:**

The online version contains supplementary material available at 10.1038/s41598-024-82344-4.

## Introduction

Precision agriculture (PA) technologies are essential for improving yield and profitability in modern agriculture. Data-driven systems play a crucial role in addressing the challenges of 21st-century food production^1^, and advancements in Agriculture 4.0 offer solutions to many on-farm challenges. However, several barriers hinder the widespread adoption of PA technologies at the farm level, including technical and equipment maintenance issues, lack of telecommunications infrastructure in rural areas, the high investment required for new technologies, and the lack of farmer-centered approaches^2^. These challenges are especially pronounced in the Hass avocado crop, as its production is concentrated in just four Latin American countries—Mexico, Colombia, Peru, and the Dominican Republic^3^—and primarily exported to developed markets with stringent requirements regarding fruit weight, shape, skin color, texture, and absence of defects^4^.

A critical challenge Hass avocado farmers face, which can be addressed using PA technologies, is irrigation scheduling. One approach is evapotranspiration modeling based on weather monitoring ^5^. Evapotranspiration refers to the total amount of water used for transpiration by the plants and evaporation from the soil^6^, and it can be estimated using meteorological data and an adjustment factor specific to each crop. For Hass avocado, experiments have shown that using 75% of evapotranspiration has led to significant yield increases^7^. On-site PA solutions for estimating water stress, aligned with farmers’ needs, are essential for effective evapotranspiration modeling. Currently, two main techniques are used: i) soil moisture in-ground sensors, and ii) hyperspectral imaging via satellites or drones. Soil moisture sensors offer benefits such as real-time data and direct soil measurements, but their limitations include low measurement accuracy, short battery life, high costs, complexity, and scalability issues for achieving high spatial resolution and coverage. Hyperspectral imaging offers detailed crop insights from a distance but suffers from low temporal and spatial resolution, complex data processing, and reliance on favorable weather conditions, especially for optical satellites and drones. This also requires specialized technical knowledge for operation and interpretation^8^.

There is a clear need for PA technologies that provide accurate, scalable, and cost-effective water stress estimation, designed with the constraints of small and medium-sized farms (SMF) in mind. Our aim in this paper is to introduce a PA solution that meets this need particularly in Colombia. To achieve this, we propose a farmer-centered four-stage methodology to develop and evaluate a solution for water stress estimation in Hass avocado crops, designed to be implemented in Colombia. The resulting solution uses novel LEO satellite connectivity for data transmission, operates off-the-grid, measures the required meteorological variables, withstands continuous exposure to the environment, and offers a superior cost-benefit ratio to the SMF compared to existing solutions. This device was the outcome of a year-long test period at an operating Hass avocado farm spanning multiple iterations and revisions to provide key insights about water stress and improve the adoption of the technology by the SMF. The collected data set for the year of testing has been freely released for further research and is available on Mendeley Data: https://data.mendeley.com/datasets/k77y7n9yhg/1.

The second section summarizes PA technology research for Hass avocado and identifies essential development areas leading to a description of the proposed methodology and its four stages. Next, the development of the solution begins with the first three stages. First, Stage 1 includes the selection of the Hass avocado case study and the identification of water stress as the main challenge. Secondly, Stage 2 follows with the desired outcomes and necessary features for the on-site PA technology solution. Thirdly, Stage 3 describes the design and implementation of the prototype. Then, the third section details the final stage, where the results of the field test are presented, and an overall assessment of the technology is made. Finally, the last section summarizes the main conclusions of the study.

## Materials and methods

### Definition of the design and evaluation protocol

The research in Hass avocado PA technologies is an emerging field^9^ with a small number of published documents^10,11^. A systematic Literature Review on agroclimatic and phytosanitary events and emerging technologies in Avocado crops^3^ concludes that although a lot of research has been done at the laboratory or in a controlled context, further research is needed to evaluate and obtain feasible solutions directly at the farms in real-world conditions.

Based on previous systematic literature reviews, a set of categories were chosen based on the type of technology that these PA solutions provide. The first category is In-ground Sensors which are sensors located at the field crop or trees, to sense any variable or parameter; the second category is Artificial Intelligence, where the solution implements algorithms or branches of this field; and the last one is Digital Images, that are images collected by satellites or drones in specific bandwidths to generate information. Table 1 includes the main research published on PA technologies for Hass avocado crop, the technologies used according with the mentioned categories, and the main challenges identified.

**Table 1:** Main research in PA technologies for Hass avocado classified as 1-In-ground sensors, 2- Artificial Intelligence and 3- Digital Images.

Technology	Devices or instruments used	Topic of research	Future research	References
1	Sensors of air humidity, temperature, precipitation, light intensity, soil temperature and moisture, Arduino platform, GPRS module	Early warning for AWC disease	Device may be designed to manage all aspects of crops and to significantly reduce costs	^12^
1	Lysimeters and digital tensiometers	Effect of constant vs. temporary water stress conducted at the Acre Experimental Station (Western Galilee, Israel)	None proposed	^13^
1	Weather stations WatchDog 2900 ET, digital caliper, digital scale, Huner lab tristimulus color, and portable colorimeter	Determination of fruit quality and its relationship with growing areas in tropical zones at the laboratory	Study the impact in quality of other interactions and management practices during the harvest and postharvest stages	^14^
1	Soil water measurement system, meteorological stations, sap flow probe, plan canopy analyzer, soil moisture probes, and light bar	Effect of soil type, fruit load and shaded areas of water use	None proposed	^15^
1,2	Soil moisture monitor ML3 Tetha Probe, weather station WatchDog serie 2000, 2450, wind monitor WindLog	Water requirements of the Hass avocado crop	Developing tools to optimize the irrigation to increase yield, using 75% of ETo	^16^
1,2	Portable spectrometer for data collection (visible – near infrared, 400–970 nm)	Early and accurate wilt disease at the Research and Education Center of University of Florida	Develop a low-cost remote sensor at UAV or helicopter	^17^
1,2	Weather stations, SMP monitoring stations	Use of SSWC for irrigation scheduling	Analyze the complexity of artificial neural networks in practice and study the application of SSWC with SAR satellites	^18^
1,3	Soil-based sensors, plant-based sensors, remote sensing (Landsat, Sentinel, and MODIS projects)	Scheduling irrigation under digital agriculture approach	Integration of remote and proximal sensing technologies using user-friendly applications	^19^
2,3	Drone images to obtain NDVI index and temperature canopy and ML algorithms	Prediction of white root rot in avocado trees using NDVI index and canopy temperature	None proposed	^20^
2,3	WorldClim data, SRTM 90 m (resolution 1 km^2^ and 8100 m^2^)	Estimation of water irrigation needs	Potential effect of non-seasonal climate variability (ENSO) and climate change on the requirements and availability of water	^21^
2,3	Sentinel-1 satellite images 10 m resolution, artificial neural network	Estimation of irrigation scheduling	Further testing and evaluation under conditions, such as La Nina, and leaf area index information needed with permanent cloudy conditions common in the Andean mountains	^22^
3	Imagery from satellites WV2 and WV3 Digital Globe, Trimble DGPS	Map yield	Apply methodology in additional growing locations and growing years	^23^
3	Moderate Resolution Imaging Spectroradiometer sensor (MODIS) for NDVI	Prediction of yield	More detailed predictions using a resolution greater than 1:100,000	^7^
3	GPS system, moderate Resolution Imaging Spectroradiometer sensor (MODIS) for NDVI	Estimate distribution of most important diseases	None proposed	^24^
3	Drones deploy for UAV digital images, Cropwat 8.0 program, and weather station	Estimation of Water Footprint as an indicator of environmental sustainability	None proposed	^25^

AWC - Avocado Wilt Complex; ENSO- El Niño–Southern Oscillation; NDVI - Normalized Difference Vegetation Index; SAR - Synthetic Aperture Radar; SMP - Soil Matric Potential; SRTM - Shuttle Radar Topography Mission; SSWC - Surface Soil Water Content; UAV - Unmanned Aerial Vehicle.

The former summary confirms that several efforts, using different devices and technologies, have been done to analyze PA technology solutions to several needs of the Hass Avocado crop, as the wilt disease detection, water stress and water requirements estimation, irrigation scheduling, and avocado quality forecasting. However, several future research topics remain, such as how to decrease the cost, a proper device design, the estimation of impact on management practices, practical technology integration and methodologies application at the field.

**Fig. 1:**
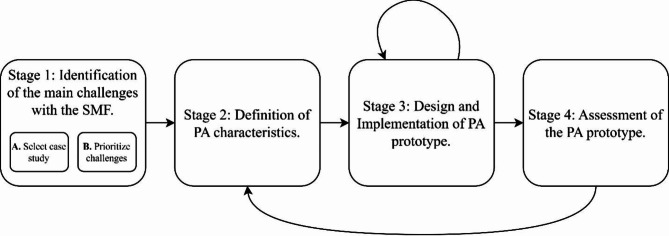
Stages of the proposed methodology. Each step is described and separated by the tasks required. Some stages involve revisions or iterations, indicated by arrows, as development advances.

Regarding the costs of the solutions, those are still economically unfeasible for small-scale agriculture in many countries^26^, and the adoption is quite low, with just 27% of the farms and ranches in the United States of America adopting a solution, concentrated in the big farms at the Midwest^27^. In Africa, less than 3%^28,29^ of the farms have a solution in place, and there are still several solutions at the piloting phase in Latin America^30,31^. There are also several other challenges such as IoT to crops, monitoring, latency in data transmission, wireless sensor networks, and smart farming network architecture^32^. Another challenge is the lack of Internet connectivity in the rural areas; in Latin America 77 million rural dwellers do not have connectivity with minimum standards (just 36.8% on average has connectivity), and for Colombia just 37,5% of rural dwellers have some connectivity, and the situation in the rural lots is even worse^33^. Due to all these difficulties, development of technological solutions for agriculture must be done according to the challenges faced by farmers to succeed. To solve this, Fig. 1 details the proposed methodology that will be followed subsequently to develop the PA solution.

*Stage 1: Identification of the main challenges to solve with the application of PA technology directly at the farms*. This stage is composed of two substages: A- Select case study and B- Prioritize the challenges. In the first substage, a case study must be selected to delimit the challenges according to crop type characteristics, the nature of its business, and the PA solutions currently applied in the crop. In the second substage, the analysis identifies the main challenges of the farmer and involves actors to use the PA solutions. To obtain those challenges a non-structured interview is executed on a farm, where the objective is to identify how the actors as farmers perceive the main problems to use the PA solutions and the value added if those problems are solved. Here, in addition to the practical evaluation directly on-site at a farm, the challenges are complemented with others identified in previous research of PA technologies.

*Stage 2: Definition of characteristics of the PA technology solution to provide answers to the challenges*. Once the challenges are identified, the methodology proceeds to define the characteristics that the PA technology solution must have to respond to those challenges. To proceed with this definition, it’s important to consider the perceived value captured in the previous step to assign priorities and define expected outcomes to choose technologies that can apply to the specific context of the case study, according to the practical on-site evaluation, and the iteration results from Stage 4, if they exist.

*Stage 3: Design and implementation of the prototype for the PA solution*. This stage includes the definition of functionality for an important use case. Functionality refers to the features and functions the prototype must have to address the desired outcome for the use case. The prototype implementation is an iteration phase where the crop-located device returns direct and indirect feedback; the direct feedback is provided by the users, while the indirect feedback is provided by the prototype itself. This stage is performed at a representative farm as identified in Stage 1.

*Stage 4: Assessment of the PA prototype*. This analysis of the final prototype iteration evaluates whether it effectively addresses the identified challenges faced by farmers. It evaluates based on indicators if each characteristic answers properly to the identified challenge at the farm. Here, an iteration process takes place to Stage 2, where a holistic evaluation is carried out, not only evaluating the technical characteristics, but how the prototype responds to the challenges posed in Stage 1.

**Fig. 2:**
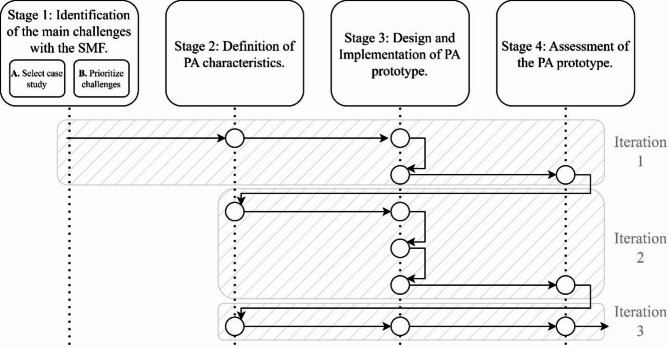
Four stages of the methodology and three iteration processes.

Three iterations were applied to the methodology execution, allowing reformulation and adding new desired outcomes that will be a key element to pose PA characteristics in Stage 2. This process places loops to refine the results obtained in Stage 4, tested at least 1 month on the selected farm. Figure 2 presents the sequence of the iteration process for each stage, considering the sub-iterations in Stage 3, which details the revision of PA solution functionality.

### Stage 1: identification of the main challenges with the SMF

First, a case study is selected based on crop type and farm typology. This work selected the case study of Hass avocado crop, due to its emerging economic importance and the big percentage of SMF involved in the cultivation. The analysis focuses on identifying the primary on-site challenges in a typical farm that a PA solution must address to result in an important impact on the SMF. The selection of the farm is based on the farm’s typology, depending on its economic and technical characteristics. Once the farm is selected, several field visits, on-site analysis and interactions with non-structured interviews to the SMF proceeded to confirm the user stories and clarify the main challenges to be solved.

The studies of typology done in 214 Avocado Hass farms of Colombia, indicates that 52% of the farms are *peasant*, 15% *commercial* and 32% are in *transition* between them. The first group have a size smaller than 9 ha, have strong limitations on market access and technical assistance; the second group have an average area of 17 ha, an average yield of 8 tons ha^-1^, with technical assistance provided by private agronomists, formal relations with commercial companies, but limitations on technical management of the crop; and the third group with an average size of 136 ha, constituted as agricultural companies, with Global GAP certifications and formal links to commercial actors, and still with difficulties to find qualified technical assistance^21^. In terms of the potential Hass avocado production zones there are a lot of zones in the Andes mountains, with average precipitation of 2,077 mm year^-1^, a range of temperature between 18.6 to 27.5 °C, and steep terrain, where there is no presence of flooded land, and 82.8%, with bimodal precipitation regime, which means two periods of high and two periods of low precipitation per year.

Based on the former attributes, a proper distance to the main municipality, to the main road, and the possibility to do several interactions with the owner and workers at the farm, a typical *transition* typology site was chosen, located in the region of Tolima with the highest yield of Hass avocado in the country^21^. The *transition* group was chosen due to its yield and profitability potential, its need for good technological management support, the presence of SMF and its adequate size for business.

**Fig. 3:**
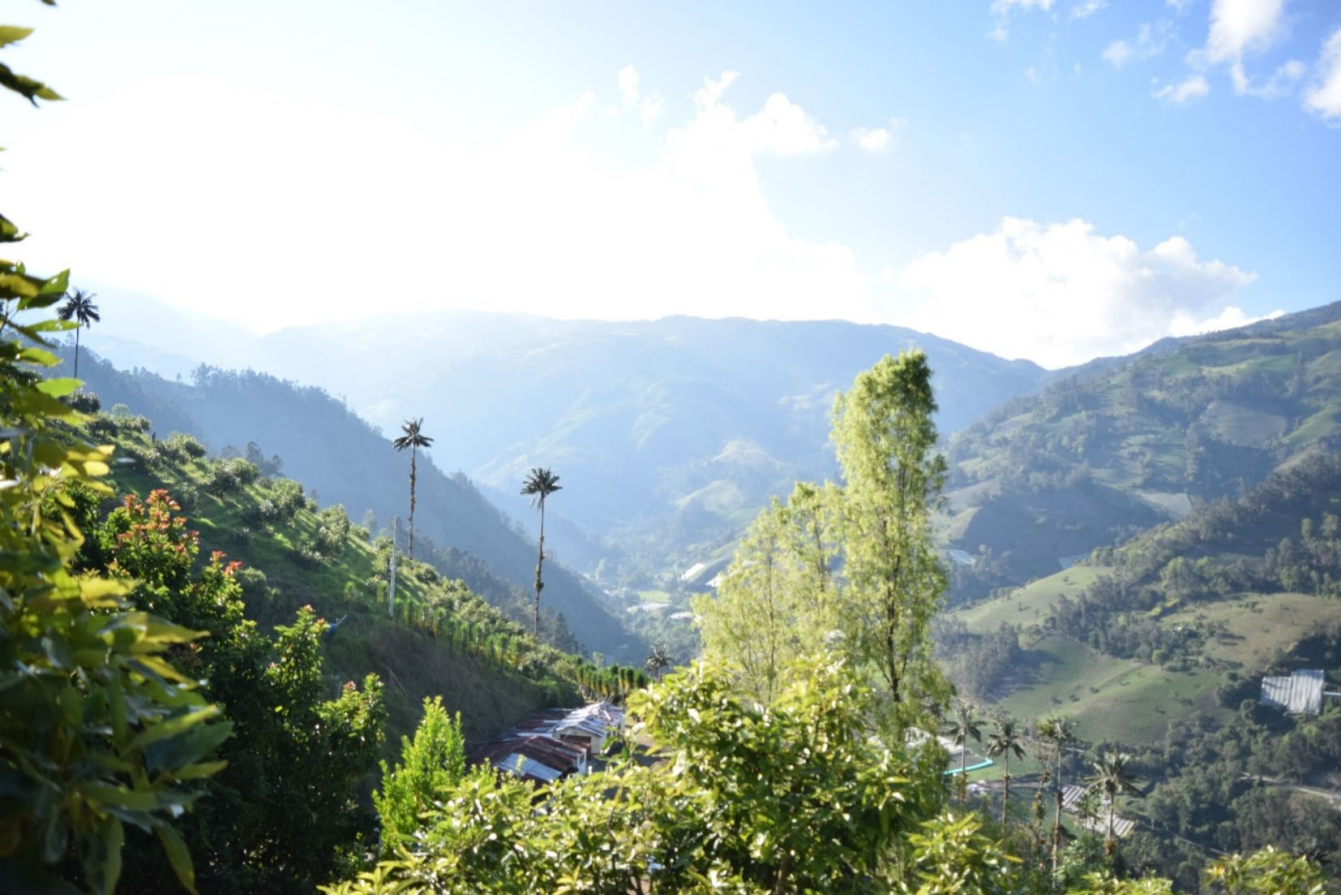
Panoramic view of the farm in front of the Anaime River canyon, whose orientation follows the global north.

The farm chosen to evaluate the prototype was “El Diamante.” located in Colombia (4°22′42.94″N, 75°29′25.25″W) in the region of Tolima, at 20 min from Anaime, a town in the Cajamarca municipality. The site is located inside the Anaime River Canyon, and it is also close to the Central Mountain range of the Colombian Andes. Figure 3 includes a panoramic view of the farm.

Based on data from the Institute of Hydrology, Meteorology, and Environmental Studies, IDEAM in Colombia, and its nearest weather station called CAJAMARCA - AUT [21215190] located 6.46 km from the farm, on the other side of the river canyon, the main data of 2022, the most recent available at the time of choosing the test site, are summarized in Table 2. The filters implemented to estimate the averages are presented in Table A.1. This farm is an appropriate evaluation and analysis site, because it represents a typical *transition* SMF Hass avocado farm in Colombia without a PA implementation, has an area of 20 ha, and a hillside location^34,35^.

**Table 2:** Main characteristics of “El Diamante” farm.

Parameter	
Location	Arrayanal, Anaime. Tolima
Latitude	4° 23′ 15.000″ N
Longitude	75° 29′ 14.086″ W
Elevation (m.a.s.l)	2100 to 2320
Slope (%)	21 to 37
Distance to municipal head (km)	12.9 km
Time from municipal head (min)	31
Annual minimum relative humidity (RH%)	52
Annual maximum relative humidity (RH%)	100
Annual average relative humidity (RH%)	88.99
Annual minimum air temperature (°C)	10
Annual maximum air temperature (°C)	22.4
Annual average air temperature (°C)	15.24
Annual precipitation (mm)**	870

**The annual precipitation in mm does not contain the information for all months because the IDEAM weather stations failed during the tested year. The months in which data are missing are October, November, and December.

**Fig. 4:**
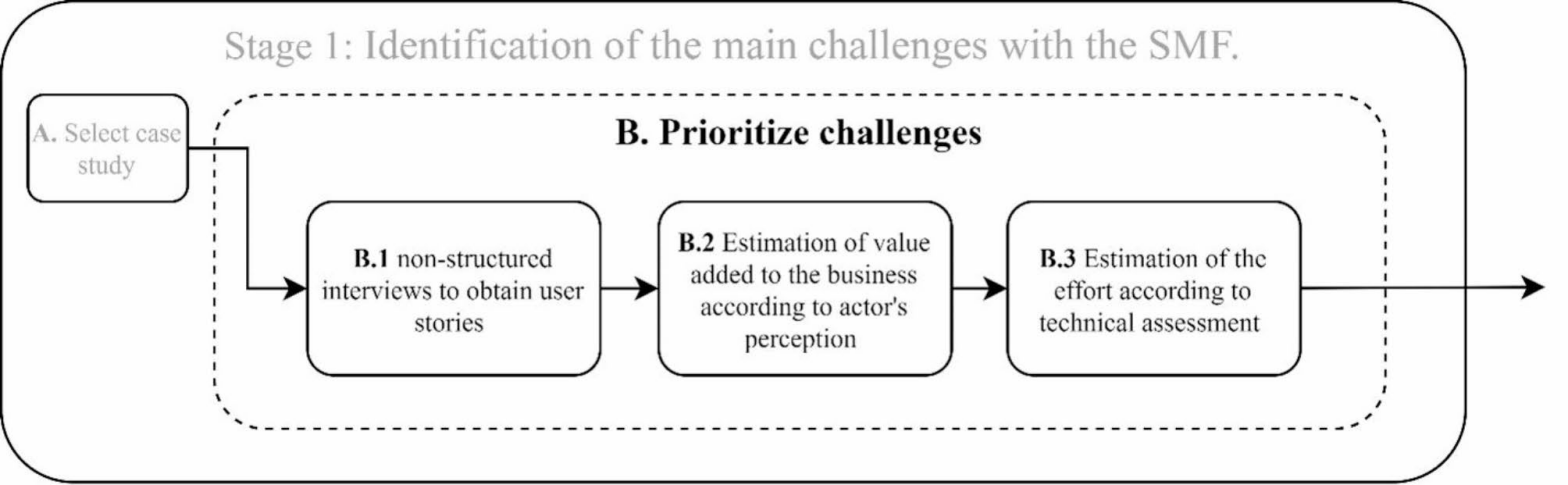
Process for prioritizing the main challenges of the SMF.

With the case study established including the crop and the typical farm for experimentation, the next step is to prioritize the main challenges for the SMF and agronomists by following the process proposed in Fig. 4. Here, the objective is to optimize the development of the solution by prioritizing work on the user stories that require the least effort but maximize the value added to the business.

**Fig. 5:**
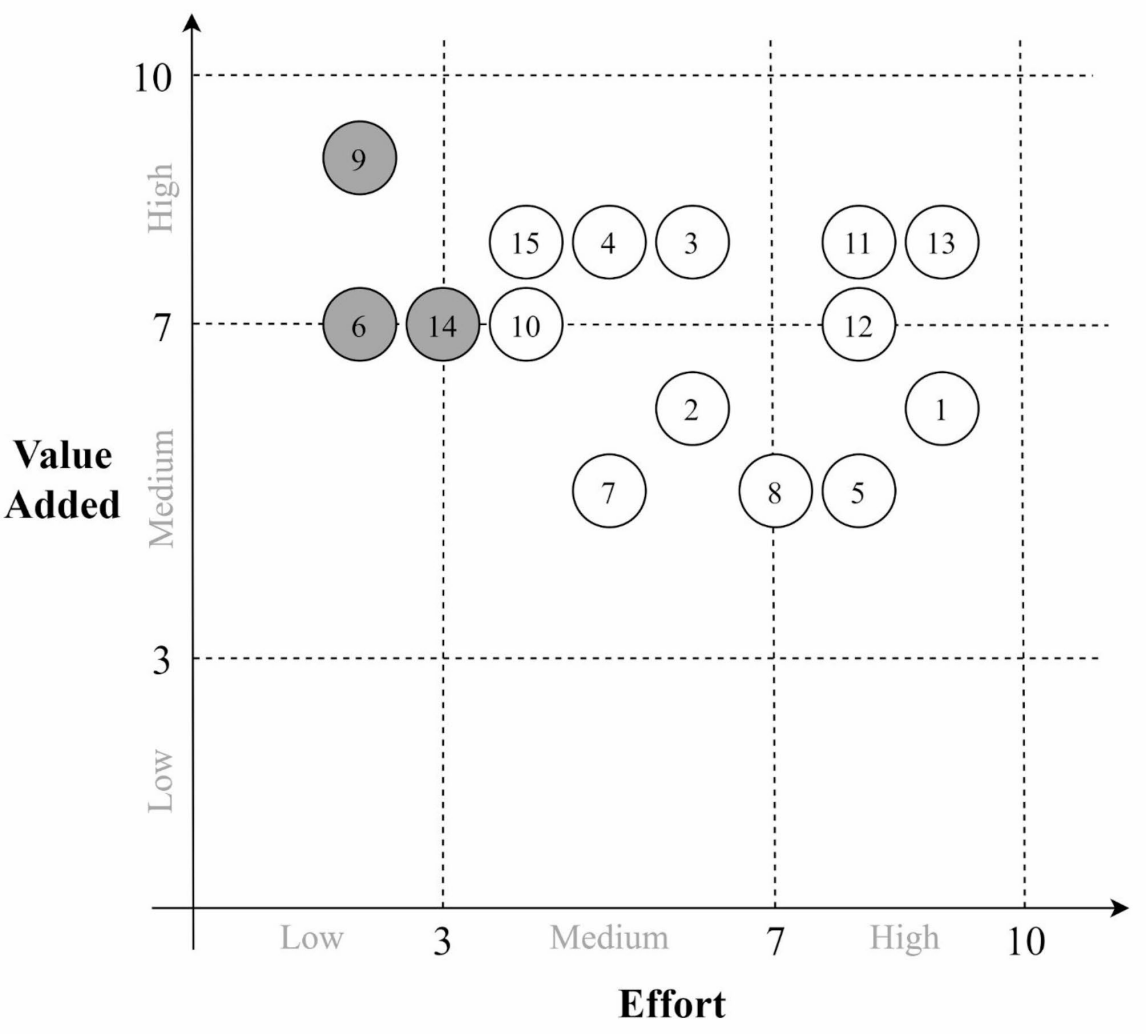
Value added vs. effort matrix of the user stories (1 to 15) collected with the non-structured interviews at the SMF and other actors of the avocado cv. Hass ecosystem. The highlighted user stories are: (6) know the temperature, humidity, solar radiation, and rainfall patterns to optimize scheduling and resource management; (9) know the presence of water stress to define irrigation scheduling; (14) measure the water requirements to show them to buyers and environmental agencies.

The farmers, private agronomists, banks, insurers, exporters and buyers of fruit were the actors related to the avocado industry included in the non-structured interviews to obtain information about the user stories and an estimation of their value added to the business. The user stories obtained are presented in Table B.1. Next, an assessment of the effort to provide a solution to each user story was done based on three aspects: collection and transmission of sensor data, complexity and availability of data storage and analysis, and the alignment of interests between the different actors of the ecosystem. The results are presented in the 2D diagram shown in Fig. 5. Here, each user story was located according to the value added to the business and the technical effort required, where a value of 1 is the lowest and 10 is the highest. User stories in the upper-left corner are of special interest, since these maximize the value added to the business while requiring the least effort.

Based on these results, the most attractive user story to solve is the water stress problem (9), since it would add the most value to the business while requiring the least effort. Additionally, both the nearest user stories of weather data monitoring (6) and estimated water use (14) are also attractive points, due to their close relationship with user story (9).

### Stage 2: definition of PA characteristics

The main challenge for the SMF and agronomist is identified in the former stage of the methodology. So, in the second stage, the most important and underserved desired outcomes for the SMF and agronomists regarding the PA solution are established. These outcomes are the base for the definition of the PA solution characteristics, which need to be aligned with the actors’ expectations. The important outcomes are those with the highest importance and the lowest satisfaction for the SMF and the agronomists.

To proceed with this stage a set of non-structured interviews were applied at El Diamante farm to the farmer and the agronomist, asking about the main outcomes that they expect to achieve with the application of a hypothetical PA technology solution at the farm, related with water stress estimation. The applied method corresponds to Outcome-Driven-Innovation® (ODI)^36^ with an iterative process in which each visit to the farm allows discovering a new series of relevant outcomes. Once the development of the prototype advances, Stage 4 checks whether the outcomes are aligned with the SMF and the agronomists’ priorities.

**Fig. 6:**
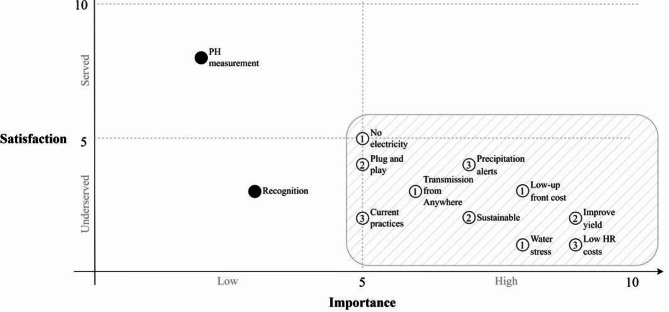
Outcomes statements of the SMF and the agronomists with the PA technology solution. Where 1 indicates the lowest importance for them and the lowest satisfaction with current solutions. Black fills in circles note desired outcomes that won’t be included in the characteristics. The number inside the circle corresponds to the order of the iterations in which that particular outcome was obtained.

The desired outcomes identified are plotted in Fig. 6, where the satisfaction dimension refers to the level in which the outcome is solved in any way by the farmer or a contracted service, and the importance dimension measures the perceived value by actors in terms of farm business. In both cases, the outcomes statements are ranked from 1 to 10. If the outcome is solved, its value must be a 10 in the satisfaction axis, and if the outcome has the highest perceived value by the actor, it must be a 10 in the importance axis. Of special interest are those outcomes located in the lower-right quadrant which correspond to the most important and most underserved.

Based on the results, Table 3 addresses the characteristics of the PA solution related each of the desired outcomes in the lower-right quadrant. Each characteristic was proposed as a possible solution to the specific outcome considering the rural context of Hass avocado.

**Table 3:** Outcomes desired and the corresponding PA technology characteristics.

Outcome type	Desired outcome	PA characteristic
Emotional and social	Being perceived by the traders as a sustainable SMF	Monitor the water usage
Consumption chain	Transmission from anywhere	Work without internet connection
	No electricity requirement	Off the grid operation
	Plug and play installation and operation at the farm	Simplicity of installation by the administrator of the farm
	Alerts provision when weather presents rapid changes and/or precipitation grows quickly	Precipitation monitoring providing alerts with adequate thresholds
	Provide water stress information and irrigation scheduling information	Methodology based on FAO or similar expertise and simplicity of information provided to the agronomist
	Adapt to local maintenance practices (prune and irrigation practices)	Operation without being affected by pruning and using current irrigation infrastructure at the farm
Financial	Improve yield and profitability	Irrigation scheduling aligned with the expert research experiments at the field
	Avoid cost of human resources at the farm	Apply irrigation only when is clearly needed
	Obtain low up-front costs	Use only the components required
	Obtain low maintenance and operating costs	Simple maintenance performed by the SMF
	Have a clear cost-benefit equation	Solution cost aligned with the profitability of the business

### Stage 3: design and implementation of the PA prototype

The initial design of the prototype serves as the foundation for the iterative design process. At this phase, the goal is not to develop an optimized solution but to generate a prototype that complies with the identified characteristics of the PA solution in the previous step, and that is flexible enough for further modifications during testing. Based on this, each characteristic from Table 3 was first translated into a requirement of the prototype as detailed in Table 4. Importantly, a requirement may implement multiple characteristics, and multiple requirements may also be needed for a particular characteristic.

**Table 4:** Prototype requirements and the corresponding characteristics they implement.

PA prototype requirement	PA characteristics implemented
Measure ETo and rainfall	Monitor the water usage Precipitation monitoring providing alerts with adequate thresholds Apply irrigation only when it is needed Methodology based on FAO or similar expertise and simplicity of information provision to agronomist Irrigation scheduling aligned with the expert research experiments at the field
Web portal for consulting information	
LEO satellite communications	Simplicity of installation by the administrator of the farm Work without Internet connection
Solar energy system	Simplicity of installation by the administrator of the farm Off the grid operation
In-house designs for complex or expensive parts	Simplicity of installation by the administrator of the farm Use only required components Simple maintenance performed by the SMF Solution cost aligned with the profitability of the business Operation without being affected by pruning and using current irrigation infrastructure at the farm

Thus, a prototype, called Cropviz, was constructed following the previous requirements. First, to define the initial set of sensors required, the reference evapotranspiration (ETo) calculation method has been defined as the FAO-56 Penman-Monteith method shown in Equation ($1$) as:

1 $$ET_{o} = \frac{{0.408\Delta \left( {R_{n} - G} \right) + \gamma \frac{{900}}{{T + 273}}u_{2} (e_{s} - e_{a} )}}{{\Delta + \gamma (1 + 0.34u_{2} )}}.$$

**Fig. 7:**
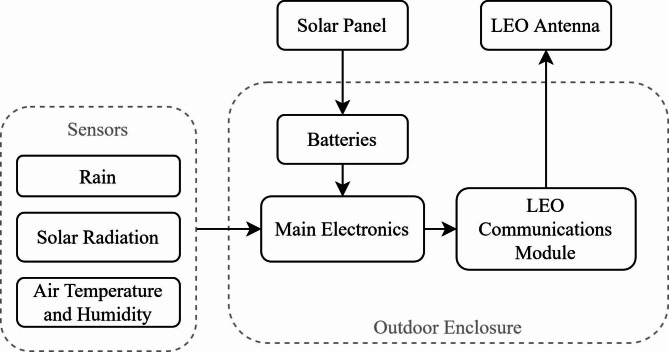
Block diagram for the Cropviz prototype, including the in-ground sensors, solar panel, battery system, main electronics, and LEO communications modules.

More details about the method can be consulted in the guidelines by the FAO^6^. Thus, measurements of air temperature, relative humidity and solar radiation are necessary for ETo estimation. Initially, wind speed measurements were not considered mandatory due to the humid weather of the farm, since variations in wind speed affect the evapotranspiration to a lesser extent than under arid conditions. In addition, a sensor for rainfall has also been added. Furthermore, solar radiation was used to estimate the sunshine duration, as was preferred by the farmer and agronomist when consulted. The sunshine duration was also calculated as detailed by the WMO, as the hours per day where the solar radiation exceeds 120 W/m^2^. Figure 7 shows the main components of the Cropviz prototype.

The solar-powered prototype operates by taking measurements from each sensor every hour and passing the data to the LEO module. This information is sent to the satellite constellation, where it is then uploaded to the internet. At this point, the user can consult the data through the web portal. No prior installation, besides the mounting, is required at the test site. Table B.2 summarizes each part of the prototype with an estimated total cost of 375.37 USD per unit.

**Fig. 8:**
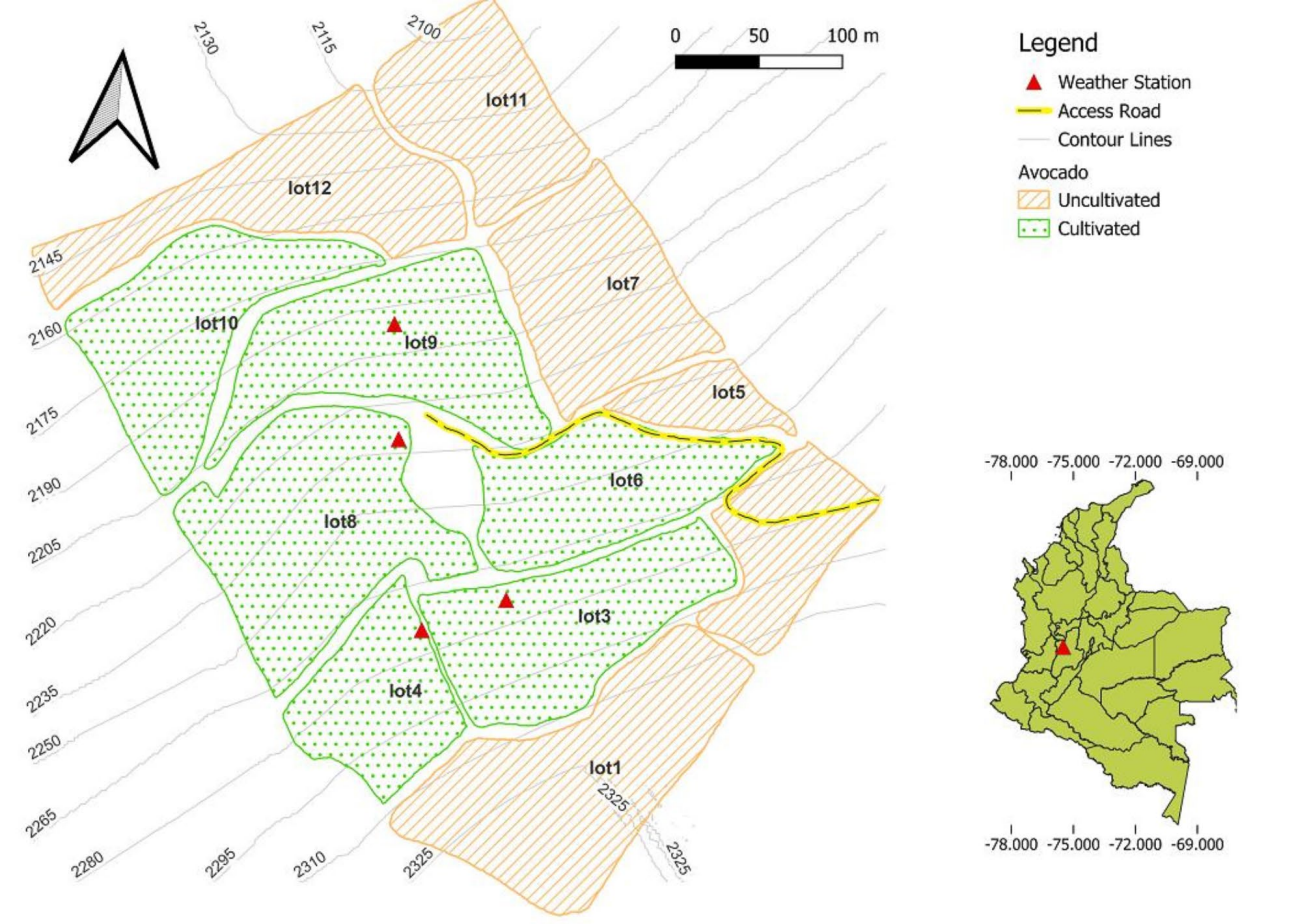
Map of the four Cropviz prototypes located at different elevations. The map base is based on Cartographic data: Imagery 2024 Maxar Technologies, Image Landsat/Copernicus Google Earth.

Now that a suitable prototype has been constructed, the next step is to begin testing at the selected farm. The first installation included three prototypes identified as Lot3, Lot8, and Lot9, at the corresponding lots of the farm in different elevations and locations to develop the test in different geographical conditions. This process began on the 27th of July. The definition of the sites of installation for these prototypes consulted the farm owner opinion to guarantee the best security, an accessible site, and no nearby obstructions, like avocado trees or energy transmission cables. A fourth prototype, identified as Lot4, was installed later in the test in January 2024 due to a request from the agronomist of the farm to collect weather data at an additional lot. Figure 8 includes a simplified map of the farm with the locations of the four prototypes. The device coordinates are shown in Table B.3.

**Fig. 9:**
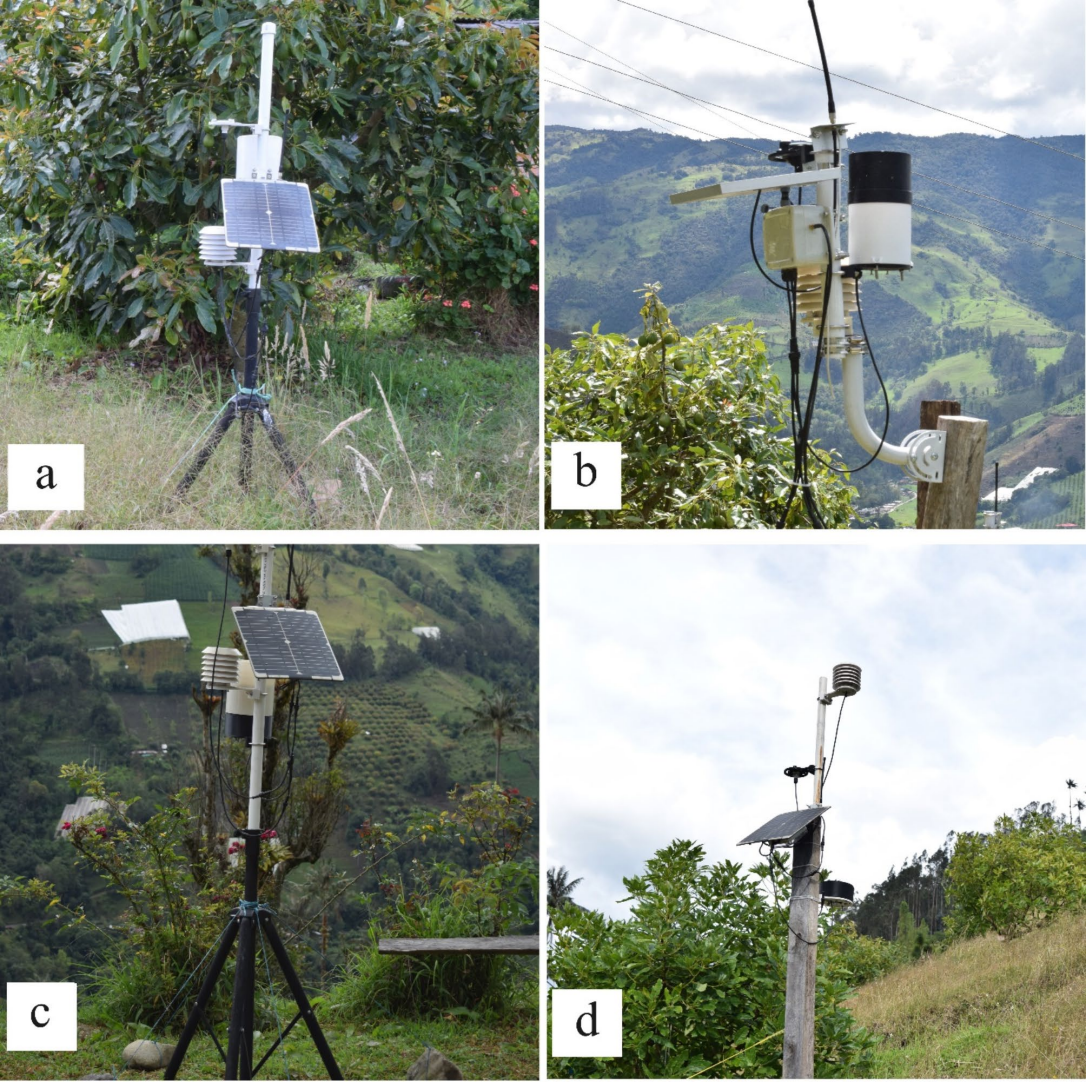
Photographs of the prototypes installed at the farm, with metal bases for the prototypes of lot 3 (**a**) and lot 8 (**c**) and the wooden bases for the prototypes of lot4 (**b**) and lot9 (**d**).

The sensors included in the prototypes were installed following the specifications of the Guide to Meteorological Instruments and Methods of Observation^37^, which indicates that the air temperature and humidity sensor should be at a height within 1.25 m to 2 m, the rain gauge must be protected against the wind, with obstacles surrounding acting as an effective windbreak for winds from all directions, and the solar radiation sensor shouldn’t be placed with reflecting obstacles above its visible horizon. The prototypes placed on Lot3, and Lot8 have a three feet metallic supporting base, supported on the ground, while the prototypes in Lot4 and Lot9 have a wooden supporting base buried in the ground. The decision about these two different methods of installation included the steep terrain conditions of each site, the availability of materials in the zone and the farmer’s ability to supervise each prototype, in terms of checking its status and location, seeking to facilitate the technological adoption of the prototype. Figure 9 includes a photograph of the four prototypes installed at the different lots.

Now that the prototypes have been installed, the farm tests begin to provide feedback about the performance of the prototype. Thus, Stage 3 becomes an iterative process where the prototype is continually improved according to the observations made during the test. Importantly, this feedback has been divided into two classes: direct and indirect. Direct feedback is provided by the users, and refers to the user experience of the current iteration of the prototype, such as ease of installation, appearance, weight, etc. Indirect feedback is provided by the prototype itself, and applies to the durability of materials, weather resistance, solar panel performance, etc. Notably, no assessment of the prototype about how it responds to the challenges from Stage 1 is made at this stage. Changes of this kind are regarded as more profound modifications to the prototype and are reserved for Stage 4, where the iterative process returns to Stage 2 to re-define the characteristics of the prototype. Consequently, 4 revisions were made on Stage 3. Table B.4 summarizes the iteration process during the test detailing the identified issues and the resulting changes.

In the following section we will present the experimental results obtained during the execution of Stage 4 of the design and evaluation protocol.

## Experimental results and discussion

The assessment of the prototype during Stage 4 evaluates whether it solves the identified challenges faced by farmers. During this analysis, two minor iterations to the prototype were first made during the test period as described in Table B.5. At the end of the test period, a more significant analysis was made using the data gathered during the year with the objective of evaluating how well the prototype addressed the main challenges identified in Stage 1.

**Table 5:** Mean and standard deviation (SD) for each of the four weather variables measured with the Cropviz prototype during the year-long test period.

Month	Air temperature (°C)	Relative humidity (%)	Sunshine duration (h/day)	Rainfall (mm)
August 2023	16.7 $\pm$ 0.7	76.6 $\pm$ 6.0	8.8 $\pm$ 1.2	2.6 $\pm$ 6.9
September 2023	16.8 $\pm$ 0.7	74.9 $\pm$ 4.4	8.5 $\pm$ 0.9	1.1 $\pm$ 4.5
October 2023	16.9 $\pm$ 0.7	80.1 $\pm$ 4.1	8.4 $\pm$ 0.8	2.2 $\pm$ 3.5
November 2023	16.6 $\pm$ 0.7	84.0 $\pm$ 3.1	8.5 $\pm$ 0.8	0.7 $\pm$ 2.1
December 2023	16.7 $\pm$ 0.7	83.1 $\pm$ 3.7	8.3 $\pm$ 1.0	*
January 2024	17.2 $\pm$ 0.8	74.6 $\pm$ 5.0	7.6 $\pm$ 1.1	0.2 $\pm$ 0.6
February 2024	17.4 $\pm$ 1.1	77.9 $\pm$ 5.8	7.9 $\pm$ 1.1	0.4 $\pm$ 1.1
March 2024	17.6 $\pm$ 1.1	79.3 $\pm$ 4.8	7.2 $\pm$ 1.7	1.1 $\pm$ 2.9
April 2024	17.1 $\pm$ 1.0	83.6 $\pm$ 7.2	7.2 $\pm$ 1.4	5.2 $\pm$ 7.1
May 2024	17.4 $\pm$ 0.7	85.9 $\pm$ 4.1	7.7 $\pm$ 1.5	8.2 $\pm$ 11.7
June 2024	16.6 $\pm$ 0.8	87.6 $\pm$ 4.0	7.8 $\pm$ 1.2	6.7 $\pm$ 10.5
July 2024	16.5 $\pm$ 0.6	83.6 $\pm$ 4.0	8.4 $\pm$ 1.0	1.4 $\pm$ 4.0

Values in the table are the Mean $\pm$ SD. *Only data for the first 5 days of December is available, which may underestimate the rainfall for the month.

First, remote sensing pilots conducted in Colombia have concluded that, even though there are clear benefits in increased productivity and reduced costs, the cost of the solutions is still too high for adoption by the SMF. This adoption barrier is vital since the farmers do not perceive a cost-effective solution^27^. In the present case, the prototype costs include initial materials for one system installed at the farm including sensors and LEO communications, the web portal, database, and cloud to receive and process the data from the farm, and the management and support for the total solution. The estimated monthly average price for the service to cover all the former cost concepts is about US 15 ha month^-1^. Further, the estimated impact of having a proper irrigation program at the farm is an increase in yield of 20% to 30%^21^ or US 250 to 375 per ha month^-1^, if the farm reaches at the seventh year a yield of 10 tons ha^-1^, which is below the average estimation for Hass avocado farms with medium technology level^7^. Therefore, the price for providing the service is about 5% of the total increase in revenues due to the increase in the yield, which is more than appropriate as a cost–benefit equation for the SMF.

**Fig. 10:**
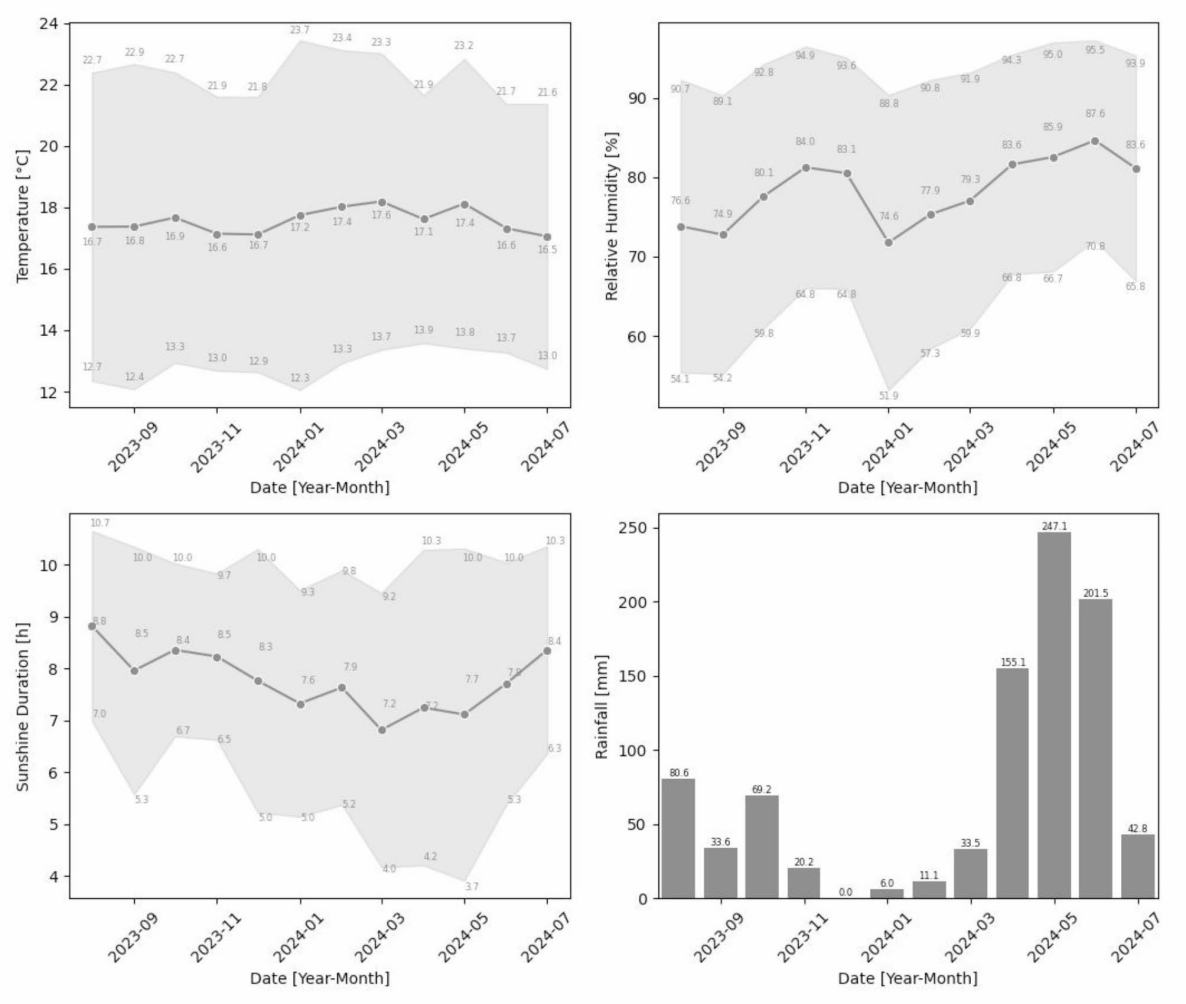
Monthly data aggregates during the test period for the 4 variables of air temperature, relative humidity, sunshine duration and rainfall. The maximum, minimum and average values are shown for each variable, except rainfall. Rainfall corresponds to the accumulated monthly value.

Secondly, Fig. 10 shows monthly data aggregates for air temperature, relative humidity, sunshine duration and rainfall gathered during the test period. For the temperature and relative humidity variables, daily maximum, minimum and average values were first calculated for each month using data averaged from all prototypes, when available. Then, the monthly maximum, minimum and average values were obtained by averaging the daily data. Secondly, the monthly maximum, minimum and average sunshine duration were determined from the daily data. Lastly, the monthly rainfall corresponds to the accumulated rainfall during the whole month. Due to rain gauge malfunctions during the first months of the test period, only rainfall data from prototype Lot3 was taken for the period of August to November. For December, only data for the first five days is available. Hence, the rainfall for the month of December may underestimate the actual value. Beginning in January, the rain gauge was replaced with an improved version and the values from the graph are an average of all prototypes during the test period.

Weather data were collected between July 28th, 2023 and July 30^th^, 2024. The first month was not considered, because only a few days were measured. Thus, approximately twelve months of testing for three of the Cropviz prototypes were conducted. The fourth prototype was installed later in the test, where data were taken from January 29^th^, 2024 to July 30^th^, 2024. Figure 10 demonstrates the capabilities of the prototype to capture weather-related data for usage by the farmer, effectively addressing user story (6). In addition, a summary of the data including mean and standard deviation values can be found in Table 5. There are many potential applications of on-site data collection, which are outside the scope of this work. However, the year-long data corresponding to the weather variables measured from the test period has been freely released to enable others to further research in the field. The data can be found on Mendeley Data by following this link: https://data.mendeley.com/datasets/k77y7n9yhg/1.

Interestingly, the test period coincided with the ENSO phenomenon, which causes lower rainfall and higher temperatures when it occurs^38^, being stronger in December-February and weaker in March-May^39^. Here, this is evidenced in the data with lower rainfall recorded since August, with the most extreme drought period in January and February (December was not considered in the analysis due to the lack of rainfall data) with very little rain and higher temperatures, followed by higher rainfall and decreasing temperatures beginning in March and more notably between April and June. The ENSO phenomenon results in negative socioeconomic and environmental consequences^40^. Therefore, the Cropviz prototype can help the farmer adapt to climate changes and mitigate the negative effects on their practice by measuring the meteorological variables and informing the user. This further reinforces the capabilities of the Cropviz prototype to solve user story (6).

Next, according to previous research in Colombia, the water stress situation occurs at least during one month per year^21^ for the Hass avocado. The yield of no-water stress treatments is higher in number of fruits, reaching more than 300 fruits per tree, compared with 200-250 in an average scenario^13^. The fruit quality is also heavily impacted by the low technological management level of the farm of the SMF^4,12^ impacting their revenues^14^. The same process detailed in previous work for Colombia^21^ was followed to estimate the irrigation water requirement (IWR) for this example to understand if and how water stress occurs and how much water would be required in the irrigation scheme. Therefore, both the Effective Precipitation (EP) and Crop Evapotranspiration ($E{T}_{c}$) have been calculated monthly, and the IWR for each month (denoted by sub-index $i$) was then found by following Equation ($2$) as:

2 $$IWR_{i} = ET_{{{\text{ci}}}} - EP_{i} .$$

For the EP, the rainfall data for each Cropviz device was first accumulated monthly. The resulting value for each month ($${P}_{i})$$ was then used to calculate the monthly EP $$(E{P}_{i})$$ by following the method proposed by the USDA Soil Conservation Service shown in Equation ($$3$$):

3 $$\begin{gathered} EP_{i} = P_{i} \cdot \frac{{\left( {125 - 0.2 \cdot {\text{P}}_{{\text{i}}} } \right)}}{{125}}\;{\text{if}}\;{\text{ }}P_{i} \le 250\frac{{{\text{mm}}}}{{{\text{month}}}}, \hfill \\ EP_{i} = 125 + 0.1 \cdot P_{i} \;{\text{if}}\;P_{i} > 250\;{\text{mm}}/{\text{month}}. \hfill \\ \end{gathered}$$

Afterwards, the monthly $$E{T}_{c}$$ was obtained by first calculating the daily (denoted by sub-index $$j$$) $$E{T}_{o}$$ value ($$E{T}_{oj})$$ using the FAO-56 Penman-Monteith method in Equation ($$1$$). Then, the daily $$E{T}_{c}$$ ($$E{T}_{cj})$$ was determined using $${K}_{c}=0.75$$ and Equation ($$4$$) as follows:

4 $$ET_{{cj}} = K_{c} \cdot ET_{{oj}} .$$

Finally, the results were added for each month using Equation ($5$) like:

5 $$ET_{{ci}} = \Sigma _{{j = 1}}^{{M_{i} }} ET_{{cj}} ,$$

where ${M}_{i}$ denotes the number of days depending on the month.

**Fig. 11:**
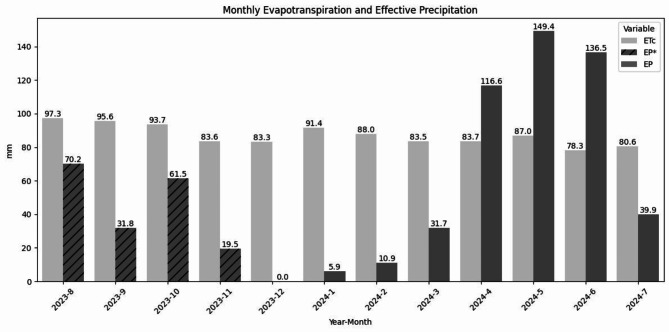
Monthly Crop Evapotranspiration and Effective Precipitation calculated. Here, irrigation is required when $E{T}_{c}$ is higher than EP. *Data from August to November was taken from only prototype Lot3. Due to rain gauge malfunctions, only data for the first 5 days of December is available, which can cause an underestimation of the actual value for the month.

Figure 11 shows both the resulting $E{T}_{c}$ and EP for the testing period following the procedure described above. As mentioned, the test period coincided with the ENSO phenomenon, causing higher evapotranspiration and lower rainfall for most months of the test period. This is reflected in the graph, where the most extreme drought period can be observed during the months of January and February with very low EP. After this, higher precipitation values were recorded for April, May and June. However, the EP was lower than the $E{T}_{c}$ for 9 of all 12 months of the test period. This indicates that the IWR is positive, meaning that the crop is undergoing water stress and would require irrigation. These results evidence that the Cropviz prototype effectively addresses the user story (9) and can identify water stress events for irrigation scheduling.

Furthermore, now that the IWR is known, the amount of water that would be applied in the irrigation scheme can be calculated, depending on the irrigation system considered. In this case, an example for a drip irrigation system has been developed following a similar procedure to a previous test pilot in Colombia^16^. First, depending on the efficiency of the irrigation system, the Gross Irrigation Water Requirement (GIWR), corresponding to the amount of water to be applied considering the efficiency of the system, can be estimated using Eq. ($6$) like:

6 $$\begin{gathered} GIWR_{i} = IWR_{i} /E\;{\text{if}}\;IWR_{i} > 0, \hfill \\ GIWR_{i} = 0\;{\text{if}}\;IWR_{i} \le 0, \hfill \\ \end{gathered}$$

where $E$ is the efficiency of the irrigation system, $GIW{R}_{i}$ is the monthly GIWR in mm and $IW{R}_{i}$ the monthly IWR from earlier in mm. Because a drip irrigation system has been considered, $E$ has been set to 0.9.

Then, the amount of water to be applied per tree per month $({V}_{i})$ can be calculated from the $GIW{R}_{i}$ and the area occupied by one tree^41^ $\left({A}_{t}\right)$ using Equation ($7$) as:

7 $$V_{i} = GIWR_{i} {\text{*}}A_{t} ,$$

where $({V}_{i})$ is the water volume in L/tree/month, and ${A}_{t}$ is the area considered in m^2^. In this example, the planting distance of 7 m from the test farm has been selected. This results in a value of ${A}_{t}=49$ m^2^.

**Fig. 12:**
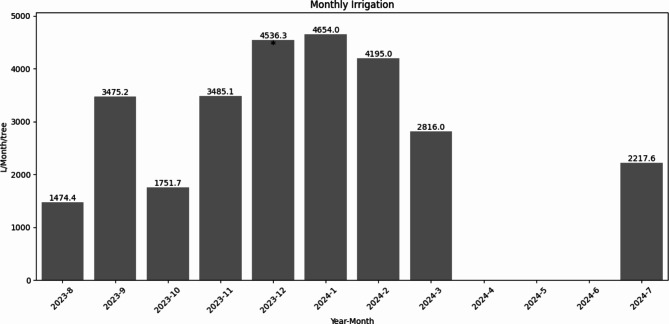
Calculated monthly irrigation per tree for each month of the test period. Negative IWR values require no irrigation. Therefore, the required irrigation is 0 for the months of April, May and June. * Data for December may overestimate the actual value due to rain gauge malfunctions.

Importantly, this is an example shown to demonstrate the potential of the Cropviz prototype to solve user story (14). Depending on the farm characteristics and the irrigation system used, these water requirements will vary. Figure 12 shows the resulting ${V}_{i}$ for the months where irrigation is required ($IWR$ > 0), using the procedure presented in this section and the data described earlier. Based on this, 9 of the 12 months require irrigation. This satisfies user story (14), as this information can be shown to buyers and environmental agencies to be better informed about the water usage at the farms. This step-by-step procedure for obtaining each variable and its relevant equations is summarized in Table 6.

**Table 6:** Procedure summary followed to obtain the water volume application considering the weather data and an irrigation system.

Daily or monthly data indicators				
Sub-index $j$		$j$	Denotes daily data	
Sub-index $i$		$i$	Denotes monthly data	
Procedure for monthly crop evapotranspiration				
1	Daily reference Evapotranspiration	$E{T}_{oj}$	Obtain $E{T}_{oj}$ from Eq. (1) using daily weather data	$\text{mm}$
2	Daily crop evapotranspiration	$E{T}_{cj}$	Calculate $E{T}_{cj}$ from Eq. (4)	$\text{mm}$
3	Monthly crop Evapotranspiration	$E{T}_{ci}$	Accumulate $E{T}_{cj}$ following Eq. (5)	$\text{mm}$
Procedure for monthly effective precipitation				
1	Monthly rainfall	${P}_{i}$	Accumulate the rainfall data for each month	$\text{mm}$
2	Monthly effective precipitation	$E{P}_{i}$	Calculate $E{P}_{i}$ from Eq. (3)	$\text{mm}$
Procedure for water volume application per tree				
1	Monthly irrigation water requirement	$IW{R}_{i}$	Get $IW{R}_{i}$ from Eq. (2)	$\text{mm}$
2	Monthly gross irrigation water requirement	$GIW{R}_{i}$	Obtain $GIW{R}_{i}$ from Eq. (6)	$\text{mm}$
3	Water volume per tree	${V}_{i}$	Calculate ${V}_{i}$ from Eq. (7)	$\frac{\text{L}}{\text{tree}}$

Finally, a features comparison between Cropviz and other PA solutions^42^ mentioned before is included in Table 7. The other solutions have been grouped into four distinct varieties and each feature has been evaluated to assess whether it has been solved by Cropviz and the other devices. This results in a point-by-point comparison between the proposed and the other existing solutions, where the advantages of Cropviz can be distinguished.

**Table 7:** Comparison between Cropviz and the PA technology solutions included in the summary.

Features	Cropviz	^12–15^	^16–19^	^20,21,23^	^7,24,25^
On-site challenges analysis	✓	x	x	x	x
On-site challenges prioritization with SMF	✓	x	x	x	x
Farmer-centered approach	✓	x	x	x	x
Field evaluation and iterations	✓	✓	✓	✓	✓
Improved cost–benefit equation for the SMF	✓	x	x	x	x
Improved up-front costs	✓	x	x	x	x
Improved management, operation and maintenance costs	✓	x	x	x	x
Works without Internet connection at the farm	✓	x	x	✓	x
Works without electricity at the farm	✓	x	x	✓	x
Simplified installation	✓	x	x	✓	x
Simplified maintenance	x	x	x	✓	x
Easy to adapt to technology evolution	✓	x	✓	✓	x
Simplified functionality	✓	✓	✓	✓	✓
Device-enabled	✓	✓	✓	x	✓
Simplified digital interface	✓	✓	✓	x	✓
Simplified customer-interface	✓	✓	✓	x	✓
Works without previous analysis or studies at the farm	✓	✓	✓	x	✓
Flexibility on aggregating other functionalities or variables	✓	x	x	x	x
Alerts provision	✓	✓	✓	✓	✓
Remote control possibility	x	x	x	x	x
Supporting several farms	✓	x	x	✓	✓
Easy to scale	✓	x	x	✓	x
Historical data visualization and analysis	✓	✓	✓	✓	✓
Extension to other crops	✓	✓	✓	✓	✓
Easy and automatic georeferentiation	✓	✓	✓	✓	✓
Adequate latency	✓	✓	✓	✓	✓
Close to real-time monitoring at the farm	✓	✓	✓	✓	✓
User friendly	✓	✓	✓	✓	✓
Different users with different profiles and authorizations	✓	✓	✓	✓	✓
Adaptability to integrate new analysis or research	✓	✓	✓	✓	✓
Accuracy in data	✓	✓	✓	✓	✓
Meeting spatial information quality	✓	✓	✓	✓	✓
Meeting required spatial resolution	✓	✓	✓	✓	✓
Current data	✓	✓	✓	✓	✓

The columns cite various PA solutions grouped by functionality.

As evidenced in the table, the Cropviz device developed following the proposed methodology has several advantages as it is farmer-centered, aligned with the on-site challenges, with low up-front and recurrent costs, has a robust cost-benefit equation for the farmer and requires no internet or electricity at the farms. Even though many improvements have been made, there continue to be multiple future avenues for work. Potential applications could be explored from the on-site data, including weather forecasting, water footprint assessment, water storage sizing, among others. Continued development of the prototype could also upgrade the capabilities of the device, reducing power consumption, simplifying maintenance and installation.

## Conclusions

The application of the four-stages farmer-centered methodology resulted in a well-aligned PA solution that solves the main challenges identified together by the farmer and the agronomists. The several iteration stages included in the methodology also play a vital role for the farmers and agronomists to be able to participate in the PA solution, and for Cropviz to assure its alignment with the practical problems at the Hass avocado farms.

The methodology required a stage of on-site assessment of a one-year period, conducted at an operating Hass avocado farm, utilizing the data gathered to determine if the characteristics of the prototype are well-aligned with the needs of the farmer. Evidently, the Cropviz prototype is an appropriate solution that can be applied in practice. However, the iterative process has not finished, and future modifications and continuous improvements are vital to deliver an even better solution. Nevertheless, the methodology clearly provides a guiding focus for development towards a successful technological solution.

The resulting PA technology solution solves the established user stories of water stress estimation using the FAO-56 equation, water usage estimation for environmental agencies and buyers, and weather monitoring for the SMF and agronomists. It measures and informs the SMF and agronomist about the drought periods where irrigation is needed and the rainy seasons where precipitation alerts can be generated. It also responds to the SMF’s cost expectations in human resources and up-front costs, respects the current practices by avoiding the installation of several in-ground sensors, is easy to install as a plug-and-play solution, transmits data from anywhere without an internet connection at the lots, and works properly off-the-grid with solar energy.

Finally, the iterations included in the methodology illustrate that the SMF and agronomists’ involvement depends on the ability to show them a practical and friendly solution on-site. This iterative process creates confidence and enables them to engage with the PA solution and provide extremely important information to improve and optimize the solution.

## Electronic supplementary material

Below is the link to the electronic supplementary material.

Supplementary Material 1

## Data Availability

All data generated during this study have been freely released and are available on Mendeley Data by following this link: https://data.mendeley.com/datasets/k77y7n9yhg/1.
