# Supplementary material for: A precision agriculture solution for water stress estimation in Hass avocado farms in Colombia
Source: Sci Rep. 2024 Dec 28;14:31178. doi: 10.1038/s41598-024-82344-4 (PMC11682183; doi:10.1038/s41598-024-82344-4)
Supplement: Supplementary file 1 — Supplementary Material 1 [file 41598_2024_82344_MOESM1_ESM.docx]

# Glossary

AWC: Avocado wilt complex

ENSO: El Niño-Southern Oscillation

EP: Effective precipitation

ETc: Crop evapotranspiration

ETo: Reference evapotranspiration

GIWR: Gross irrigation water requirement

IWR: Irrigation water requirement

LEO: Low-Earth Orbit

NDVI: Normalized Difference Vegetation Index

ODI: Outcome-Driven-Innovation®

PA: Precision Agriculture

SAP: Soil Matric Potential

SAR: Synthetic Aperture Radar

SMF: Small and medium-sized farms

SRTM: Shuttle Radar Topography Mission

SSWC: Surface Soil Water Content

UAV: Unmanned Aerial Vehicle

# Appendix A

## A.1 Farm Characteristics

The farm has an elevation difference of approximately 220 meters (about 721.78 ft) from the lowest terrain, located near 2100 m.a.s.l to the highest terrain near 2320 m.a.s.l, making it a hillside crop, very common for Hass avocado in Colombia. The farm is composed of twelve (12) lots (lot1 through lot12) located at different altitudes.

At the farm, 2,000 Hass avocado trees have been grown since 2019, having an age of 4 to 5 years, a height of approximately two (2) meters, and distributed into five lots: lot 3, lot 4, lot 6, lot 8, lot 9, and lot 10. Other lots did not contain avocado trees. According to Thornthwaite Climate Classification, this farm is classified as B2 humid (40 – 59.9) of moisture index. The site has tropical weather, that applies to 82.8% of the Hass avocado farms in Colombia, presenting a bimodal precipitation regime caused by its location in the Magdalena-Cauca basin, with two periods of high precipitation (April-May and October-November), and two periods of drought (January-February and July-August).

**Table A.1** Parameters to estimate the weather variables averages.

|  | Temperature | Relative Humidity | Rainfall |
| --- | --- | --- | --- |
| Initial date | 01/01/2022 | 01/01/2022 | 01/01/2022 |
| Final date | 30/12/2022 | 30/12/2022 | 30/12/2022 |
| Time and Frequency Series | Standard | Standard | Standard |
| Parameter | TEMPERATURE | RELATIVE HUM | PRECIPITATION |
| Complete list | Air Temperature at 2 meters annual maximum | Relative air humidity at 2 meters annual maximum | Total Hourly Precipitation |
|  | Air Temperature at 2 meters annual minimum | Relative air humidity at 2 meters annual minimum |  |
|  | Air Temperature at 2 meters annual average | Relative air humidity at 2 meters annual average |  |
| Department | Tolima | Tolima | Tolima |
| Municipality | Cajamarca | Cajamarca | Cajamarca |
| Weather Station | CAJAMARCA - AUT [21215190] | CAJAMARCA - AUT [21215190] | CAJAMARCA - AUT [21215190] |

# **Appendix** B

## B.1 User stories

**Table B.1** User stories derived from the field work with the actors of the Hass ecosystem. Where 1 represent the lowest estimation of value added to the business according to the actor’s perception, and 10 the highest estimation of value.

| **Actor** | **User story #** | **User story** | **Effort (1-10)** | **Value added**  **(1-10)** |
| --- | --- | --- | --- | --- |
| Bank/insurer | 1 | I need to estimate the crop risk to provide or not a loan or an insurance | 9 | 6 |
|  | 2 | I need to know the production to estimate the amount of loan | 6 | 6 |
| Exporter | 3 | I need to know the amount and quality of production to define the price per kilo | 6 | 8 |
|  | 4 | I need to know when the pesticides were applied to ensure the health compliance | 5 | 8 |
|  | 5 | I need the farm to have traceability | 8 | 5 |
| Agronomist | 6 | I need to know the temperature, humidity, solar radiation and rain patterns to schedule the fertilizers | 2 | 7 |
|  | 7 | I need to know the presence of pollinators to decide if their cultivation is required at the farm | 5 | 5 |
|  | 8 | I require to know if pest arrival is close to define the pesticides application | 7 | 5 |
|  | 9 | I need to know the presence of water stress to define irrigation scheduling | 2 | 9 |
| Farmer | 10 | I need to show the crops advance to get enough capital | 4 | 7 |
|  | 11 | I need to forecast production to tell the exporter how much quantity to be sent | 8 | 8 |
|  | 12 | I require a robust forecasting to improve price per kilo | 8 | 7 |
|  | 13 | I need to know the causes of bad quality, to reduce the rejection rate | 9 | 8 |
|  | 14 | I need to measure the water requirements to show them to buyers and environmental agencies | 3 | 7 |
|  | 15 | I need to know the tree’s health to define fertilizers scheduling | 4 | 8 |

## B.2 Prototype summary

**Table B.2** Summary of each part of the Cropviz prototype. The prices use a USD-COP conversion of 3825.92. * The SHT31 sensor was switched to the SHT45 variant starting April 30th to use the most recent version. Pricing does not change considerably.

| Part | Part Number | Additional information | Price (USD) | Price (COP) |
| --- | --- | --- | --- | --- |
| Main electronics + Antenna + Solar Panel | Developed in-house | Obtains sensor readings  Communicates with the LEO module Measures battery and solar panel voltage and currents | 228.46 | 874,070 |
| LEO Module | Swarm Technologies M138 Tile | Transmits to the LEO constellation | 80 | 306,074 |
| Air Temperature and Humidity Sensor | Sensirion SHT31-DIS-F * | Sensor is housed inside a passively ventilated radiation shield | 18.87 | 72,195 |
| Solar Radiation Sensor | ams Osram TSL2591 | Calibrated with an Apogee SP-110 pyranometer | 27.18 | 103,989 |
| Rain Gauge | Developed in-house | 0.2 mm steps | 20.86 | 79,809 |

## B.3 Prototype coordinates

**Table B.3** Prototype coordinates in decimal degrees.

| Prototype | Latitude | Longitude |
| --- | --- | --- |
| Lot3 | 4°22'39.88"N | 75°29'24.06"W |
| Lot8 | 4°22'43.01"N | 75°29'26.15"W |
| Lot9 | 4°22'45.25"N | 75°29'26.23"W |
| Lot4 | 4°22'39.3"N | 75°29'25.7"W |

## B.4 Prototype issues identified

**Table B.4** Each identified issue and resulting change to the prototype corresponding to Iterations 1 and 2 are explained. There were no revisions during Iteration 3.

| Revision | Identified issue | Changes |
| --- | --- | --- |
| 1 | Security concerns about the prototype | Add an adjustable mounting bracket that can be secured to wooden bases or poles that can be installed with the tools available at the farm at secure sites |
|  | Difficulties leveling the supporting base as required for the prototype |  |
|  | Material availability for the supporting base |  |
|  | Strong winds can blow over the prototype |  |
| 2 | The tin-plated contacts of the sensor circuit board connectors corrode and cause malfunctions | The circuit board contacts exposed to the environment were switched to gold-plated variants |
| 3 | The sensor circuit boards corrode and cause malfunctions | The sensor circuit boards exposed to the environment were covered with conformal coating |

## B.5 Prototype minor revisions

**Table B.5** Minor iterations to the prototype detailing the changes made.

| Iteration | Challenge not being met | Changes |
| --- | --- | --- |
| 1 | Agronomists expressed interest in measuring wind speed in certain parts of the farm | Add an anemometer to measure wind speed to the prototype at Lot9. |
| 2 | Dust, dirt, leaves and other debris can block the rain gauge funnel and enter the internal mechanism, causing malfunctions | A separate mesh part was added to the top of the rain gauge to block spiders and insects from entering and make cleaning of leaves and big insects easier by taking out this part and manually removing them. |
|  | Spiders and insects can enter the rain gauge and block the internal mechanism and cause malfunctions. This effect was more prevalent in certain lots. |  |
|  | The rain gauge is difficult to 3D print consistently, impacting the iteration process. | A re-design of the rain gauge was made to reduce its size. |
